# Multiple scattering in grazing-incidence X-ray diffraction: impact on lattice-constant determination in thin films

**DOI:** 10.1107/S1600577516003672

**Published:** 2016-04-20

**Authors:** Roland Resel, Markus Bainschab, Alexander Pichler, Theo Dingemans, Clemens Simbrunner, Julian Stangl, Ingo Salzmann

**Affiliations:** a Institute of Solid State Physics, Graz University of Technology, Graz, Austria; b Faculty of Aerospace Engineering, Delft University of Technology, Delft, The Netherlands; c Institute of Semiconductor and Solid State Physics, Johannes Kepler University, Linz, Austria; d Institute of Solid State Physics, University of Bremen, Bremen, Germany; e Institute of Physics, Humboldt University, Berlin, Germany

**Keywords:** organic thin films, X-ray scattering, grazing-incidence diffraction, surface reflection, X-ray refraction, grazing-incidence X-ray diffraction, refraction correction, thin films

## Abstract

Dynamical scattering effects are observed in grazing-incidence X-ray diffraction experiments using an organic thin film of 2,2′:6′,2′′-ternaphthalene grown on oxidized silicon as substrate. Here, a splitting of all Bragg peaks in the out-of-plane direction (*z*-direction) has been observed, the magnitude of which depends both on the incidence angle of the primary beam and the out-of-plane angle of the scattered beam. The incident angle was varied between 0.09° and 0.25° for synchrotron radiation of 10.5 keV. This study reveals comparable intensities of the split peaks with a maximum for incidence angles close to the critical angle of total external reflection of the substrate. This observation is rationalized by two different scattering pathways resulting in diffraction peaks at different positions at the detector. In order to minimize the splitting, the data suggest either using incident angles well below the critical angle of total reflection or angles well above, which sufficiently attenuates the contributions from the second scattering path. This study highlights that the refraction of X-rays in (organic) thin films has to be corrected accordingly to allow for the determination of peak positions with sufficient accuracy. Based thereon, a reliable determination of the lattice constants becomes feasible, which is required for crystallographic structure solutions from thin films.

## Abstract 2

The use of grazing-incidence X-ray diffraction to determine the crystal structure from thin films requires accurate positions of Bragg peaks. Refraction effects and multiple scattering events have to be corrected or minimized.

## 1. Introduction   

Thin films of ordered organic assemblies prepared on isotropic substrates play a key role in a number of areas of fundamental and application-related science such as organic electronics (O’Neill & Kelly, 2011 ▸; Katz & Huang, 2009 ▸) but also emerging fields, *e.g.* the investigation of substrate-mediated polymorphs in pharmaceutical research (Werzer *et al.*, 2014 ▸). In all of these areas of research, crystallographic investigations are performed on molecular crystals, polymers and liquid crystals prepared either from solution or the gas phase (Liscio *et al.*, 2012 ▸; Cataldo & Pignataro, 2013 ▸; Chattopadhyay *et al.*, 2014 ▸). In this context, grazing-incidence X-ray diffraction (GIXD) is certainly the most valuable experimental technique for assessing (ultra) thin-film structures, as it allows mapping of large areas in reciprocal space in order to deduce information on polymorphism, preferred orientation, mosaicity and both vertical and lateral crystal sizes (Schiefer *et al.*, 2007 ▸; Resel, 2008 ▸; Salzmann *et al.*, 2008 ▸; Rivnay *et al.*, 2012 ▸). Particular interest lies in investigating new polymorphs, which frequently occurs when crystallization processes take place in the presence of a substrate (Wedl *et al.*, 2012 ▸). The solution of such polymorphs, however, still remains challenging, as no universal procedure has been developed so far (Salzmann *et al.*, 2011 ▸; Mannsfeld *et al.*, 2011 ▸; Krauss *et al.*, 2008 ▸; Nabok *et al.*, 2007 ▸). Apart from fully theoretical approaches like attempts to predict crystal structures directly from the chemical formulae of the constituents (Della Valle *et al.*, 2008 ▸), the first step towards crystal structure determination of (ultra) thin films is deriving the unit-cell parameters on the basis of the experimental Bragg peak positions of GIXD data. Subsequently, the molecular packing is either determined from the experimental peak intensities (Schiefer *et al.*, 2007 ▸; Mannsfeld *et al.*, 2011 ▸; Salzmann *et al.*, 2012 ▸; Pichler *et al.*, 2014 ▸) or by theoretical modelling (Nabok *et al.*, 2007 ▸; Salzmann *et al.*, 2011 ▸) with the experimental data used to evaluate the quality of the result.

GIXD experiments on thin films require a detailed understanding of the scattering processes involved and are connected with sophisticated data treatment for extracting the desired information on the thin-film structure. As largely disregarded in pertinent literature, in such experiments multiple-scattering events can arise from a combination of X-ray beams optically reflected at the interfaces involved (*e.g.* substrate/thin film) with those scattered by the film. As a consequence, additional features in the diffraction pattern can appear which might be misleading or, at least, difficult to interpret. In this context, related experimental observations are reported and theoretically well described for grazing-incidence small-angle X-ray scattering (GISAXS) experiments (Rauscher *et al.*, 1999 ▸; Lee *et al.*, 2005 ▸, 2008 ▸; Busch *et al.*, 2006 ▸; Stein *et al.*, 2007 ▸). In the case of GIXD, however, fewer reports exist on multiple-scattering events, although the peak positions can be significantly influenced by refraction effects of the X-rays (Breiby *et al.*, 2008 ▸; Toney & Brennan, 1989 ▸). The theoretical treatment of X-ray scattering from thin films taking into account multiple scattering from surfaces can be performed using the distorted wave Born approximation (Daillant & Alba, 2000 ▸; Lazzari, 2009 ▸).

Within the present work, we report on Bragg-peak splitting observed in synchrotron GIXD experiments on a thin film of the organic semiconductor 2,2′:6′,2″-ternaphthalene, which were carried out with the aim of solving its crystal structure from a thin film (Pichler *et al.*, 2014 ▸). The origin of peak splitting is described, its impact is assessed, the required corrections for data treatment are given, and experimental approaches to avoid such a splitting of Bragg peaks in future GIXD experiments are discussed. We demonstrate that these effects need to be considered in practical applications of GIXD due to their significance and their crucial impact on the accuracy of experimental thin-film structure solution procedures.

## 2. Experimental details   

A thin film of 2,2′:6′,2″-ternaphthalene (NNN) of extraordinary high structural quality comprising crystalline islands with an average height of ∼100 nm and a lateral width in the micrometer range (Pichler *et al.*, 2014 ▸) was prepared by physical vacuum deposition using hot-wall epitaxy (Sitter *et al.*, 2003 ▸) on thermally oxidized silicon wafers (SiO_*x*_). The root-mean-square roughness of the substrate (σ_RMS_) was determined by atomic force microscopy to be ∼0.2 nm, with that of the NNN film being σ_RMS_ ≃ 30 nm (Pichler *et al.*, 2014 ▸). As the SiO_*x*_ surface is both isotropic and amorphous, the NNN molecules grow uniaxially aligned and form a fiber-textured film with a fiber axis perpendicular to the substrate surface. Such films can be regarded as a two-dimensional powder, because no in-plane alignment of the crystallites exists. The crystal structure of NNN, as solved by GIXD, is characterized by a layered herringbone arrangement of the molecules with a monoclinic unit cell of *a* = 0.8148 nm, *b* = 0.5978 nm, *c* = 1.945 nm and β = 94.6° (Pichler *et al.*, 2014 ▸). The NNN crystals are oriented with the 001 plane parallel to the substrate surface and show a mosaicity of 0.06°. A recent crystal structure solution *via* single-crystal diffraction reveals essentially identical lattice constants for *a* = 0.81498 nm and *b* = 0.59817 nm, but a small deviation for the lattice constant *c* = 1.96891 nm and the monoclinic angle β = 94.397° (Moret, 2015 ▸).

**Figure 1:**
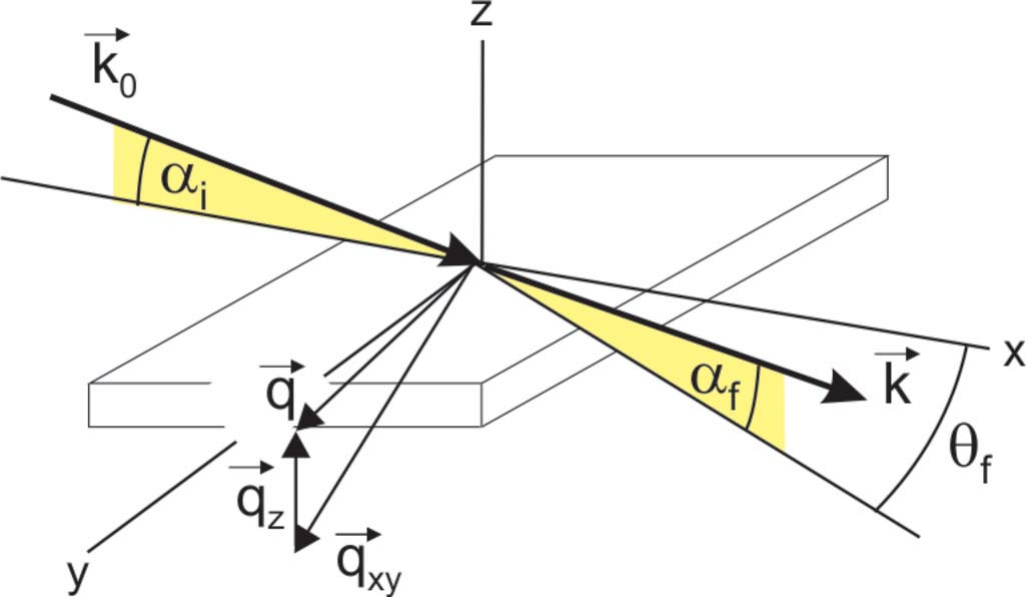
Experimental geometry for grazing-incidence X-ray diffraction with *k*_0_ and *k* as wavevectors of incoming and scattered beams, respectively; α_f_ and θ_f_ are the scattering angles, **q** is the scattering vector represented by an in-plane component *q*_*xy*_ and an out-of-plane component *q*_*z*_.

GIXD investigations were performed at beamline W1 at DESY-HASYLAB (Hamburg, Germany) using a primary-beam energy of 10.5 keV (wavelength 0.11808 nm). Using pseudo 2 + 2 geometry, the incidence angle of the primary beam (α_i_) was varied between 0.09° and 0.25° in steps of 0.01°. Data were recorded by varying the in-plane scattering angle (θ_f_) between 6° and 36° (step width 0.04°, 2 s integration time per step) employing a one-dimensional position-sensitive detector (Mythen 1K, Dectris) mounted in the *z*-direction. One single step covers an angular range of out-of-plane scattering of Δα_f_ ≃ 3.8°; a detailed sketch of the experimental geometry is given in Fig. 1 ▸. Two-dimensional representations of the experimental data were plotted with the custom-made software *PyGID* using a logarithmic color scale (Moser, 2012 ▸). In order to represent Bragg peaks as a function of α_f_, the peaks were integrated along θ_f_ in a range of 1° around the peak maxima. The individual peak parameters were obtained by independently fitting the data twice with the respective software packages *Origin 9.1* (using Voigt functions for peak positions and peak widths) and *Fityk* [using Gaussian functions for the peak area (Wojdyr, 2010 ▸)]. Error analysis was performed on the basis of α_f_ taking the full width at half-maximum (FWHM) as error margin of the peak position. The experiments were performed under a helium atmosphere using the dome of the DHS900 attachment (Resel *et al.*, 2003 ▸) to reduce sample degradation (Neuhold *et al.*, 2012 ▸).

## 3. Results and discussion   

**Figure 2:**
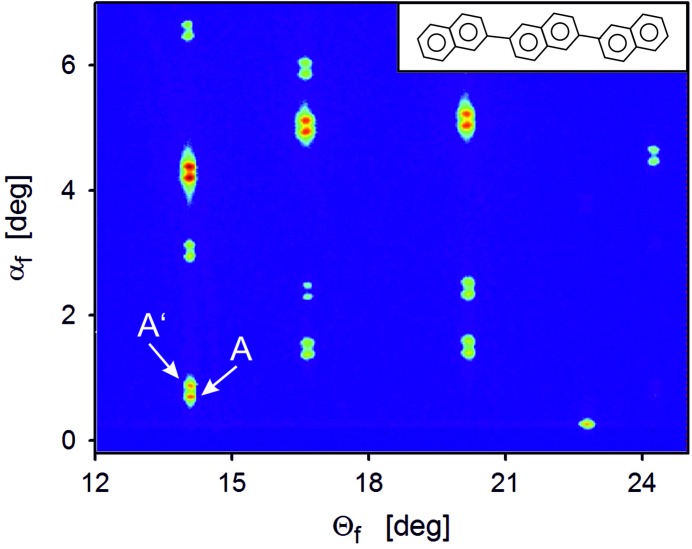
Grazing-incidence X-ray diffraction pattern of a ternaphthalene thin film on an isotropic silicon oxide surface recorded at α_i_ = 0.15°; intensities are plotted on a logarithmic color scale. The peaks A and A′ represent the components of the split 110 Bragg peak. The inset gives the chemical structure of the molecule.

Fig. 2 ▸ shows the result of GIXD investigations performed with an incident angle α_i_ = 0.15°. The measured intensities are plotted as a function of the in-plane scattering angle θ_f_ and the out-of-plane scattering angle α_f_. Surprisingly, a doubling of all Bragg peaks is observed over the whole diffraction pattern. Apart from the Bragg peaks, the so-called ‘Yoneda peak’ is visible in the map as a weak horizontal line with a peak maximum at α_f_ = 0.147°, which is known to coincide with the critical angle of total external reflection α_C_ (Yoneda, 1963 ▸). As α_C_ depends both on the X-ray wavelength and on the total electron density of the scattering material, for the present case, two contributions, one from the organic adsorbate and one from the inorganic substrate, are expected. On the basis of the known crystal structure of NNN, its electron density is determined to 397 nm^−3^ which yields a refractive index of *n*_org_ = 1 − δ with δ = 2.48 × 10^−6^ and, finally, α_C,organic_ = (2δ)^1/2^ = 0.128°. The critical angle of the amorphous silicon oxide substrate is determined on the basis of electron densities obtained from X-ray reflectivity investigations (Neuhold *et al.*, 2012 ▸) to α_C,substrate_ = 0.166° for the present X-ray wavelength. The observed peak maximum of the Yoneda peak lies exactly between these two values; however, the experimental setup does not allow separating the two contributions due to limited resolution.

**Figure 3:**
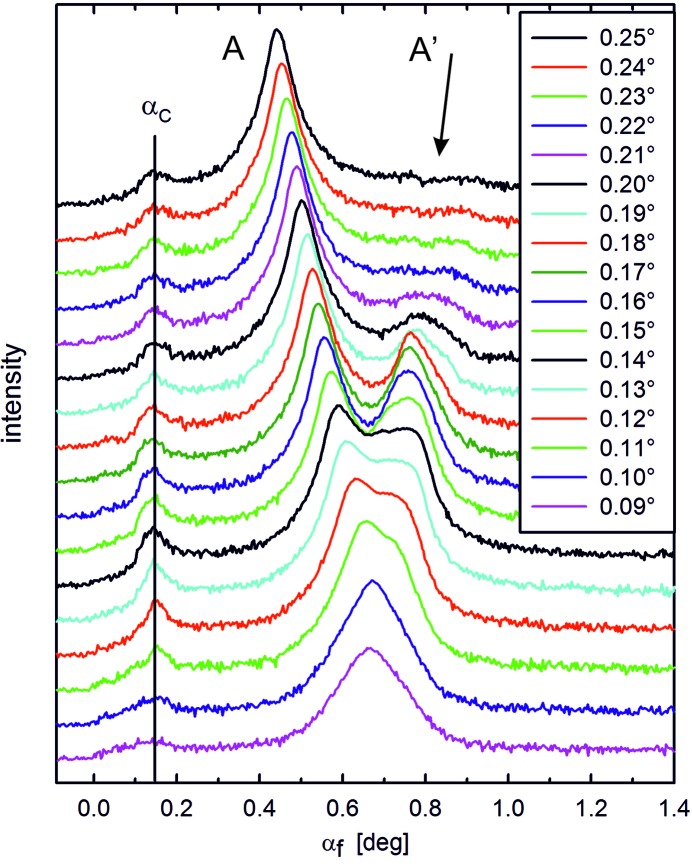
Line scans obtained by integration of reciprocal space map data along θ_f_ across the split 110 Bragg peak (integration range 1°). Data are collected at different incidence angles α_i_ of the primary beam covering a range of 0.09° to 0.25°; curves are vertically shifted for clarity. The average position of the Yoneda peak is given by a vertical line labelled as α_C_.

**Figure 4:**
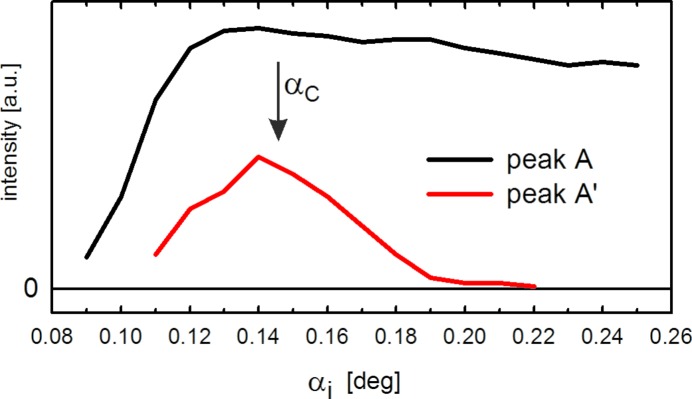
Integrated peak intensities of the peaks A and A′ as a function of the incident angle of the primary beam α_i_. The position of the observed critical angle of total external reflection α_C_ is marked with an arrow.

In a next step, the split 110 peak (components denoted as A and A′ in Fig. 2 ▸) was recorded for different incident angles α_i_ in the range between 0.09° and 0.25°. The results are shown in Fig. 3 ▸ with the peak intensities plotted as a function of the out-of-plane scattering angle α_f_. For this range of α_i_ we observe a gradual transition between one single peak for low values, over split peaks for intermediate α_i_, back to one dominant single peak. For low angles (α_i_ < 0.14°), the two peaks are difficult to resolve, while for large angles (α > 0.20°) the intensity of the higher α_f_ peak (A′) diminishes strongly. The respective intensities of the peaks are plotted separately in Fig. 4 ▸: in the case of peak A, the intensity strongly increases up to α_i_ = 0.14° and decreases slightly for larger angles. For peak A′, however, the intensity shows a distinct maximum for α_i_ = 0.14°, which is around the experimentally observed value of α_C_. Consequently, this finding already qualitatively suggests that the observed peak splitting is correlated with the reflection of the incident X-rays at the substrate surface that is maximal at α_C_.

The phenomenon of peak splitting as observed here can, indeed, be rationalized by multiple-scattering events, where Bragg scattering from the organic crystallites occurs in combination with the optical reflection of X-rays from the substrate surface (Rauscher *et al.*, 1999 ▸; Lee *et al.*, 2008 ▸). In the following, a qualitative description is given first. Peak A arises solely from direct Bragg scattering of the incident primary beam on the crystalline organic film, which is the information typically headed for in GIXD experiments on thin films. Finding the maximum intensity of peak A around α_C_ (*cf*. Fig. 4 ▸) follows from the enhancement of the transmission function (Dosch, 1992 ▸; Als-Nielsen & McMorrow, 2011 ▸). In contrast, the intensity maximum of peak A′ arises from a two-step process: first, the primary beam is optically reflected at the interface between the organic film and the substrate, and, second, subsequent Bragg scattering of this primary beam portion takes place at the organic crystallites. The marked decrease in peak intensity of A′ observed for α_i_ > α_C_ is due to the strong decrease in the reflectivity of the primary beam at the film/substrate interface above α_C_ (Als-Nielsen & McMorrow, 2011 ▸), which dominates over effects of the shorter X-ray path lengths at higher scattering angles by reducing absorption. For α_i_ < α_C_, that is, for the region of total external reflection of the primary X-ray beam, the increasing footprint of the primary beam at the sample surface causes an increase in the intensity of the totally reflected beam. Note that the NNN film under investigation exhibits a highly corrugated morphology [see Pichler *et al.* (2014 ▸) for corresponding scanning force microscopy data]. Therefore, in the present case, the attenuation of the primary beam below α_C_ is much less effective than for an ideal flat film and reflection at the substrate can efficiently occur.

**Figure 5:**
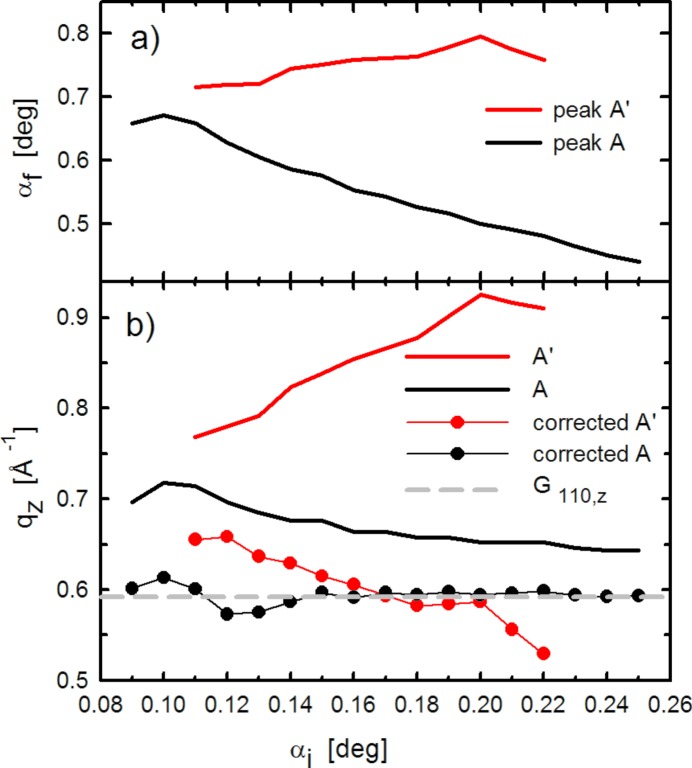
(*a*) Positions of the peaks A and A′ given by the out-of-plane scattering angle α_f_ as a function of the incidence angle of the primary beam α_i_. (*b*) Calculated contribution of the scattering vector in the direction perpendicular to the surface (*q*_*z*_) for peaks A and A′ without (lines) and with corrections for both the inherent scattering path and optical refraction of X-rays (lines + symbols). For comparison, the *q*_*z*_ value of the reciprocal lattice vector *G*_110,*z*_ is given.

Turning now to a more quantitative description of the phenomenon, the precise positions of peaks A and A′ as fitted with two Voigt functions are taken into account (Fig. 5*a* ▸). Note that for the data recorded at lowest and highest values of α_i_ we refrain from providing the peak positions in Fig. 5(*a*) ▸, as the peaks are not sufficiently separated for α_i_ < 0.11° and the intensity of peak A′ is too low for α_i_ > 0.20° to precisely determine α_f_. For a simple treatment of the peak splitting it is convenient to transform the experimental results into reciprocal space. The total scattering vector (**q**) in our experiment is represented by an in-plane (*q*_*xy*_) and an out-of plane component (*q*_*z*_), where the *xy* plane is the substrate surface with the *z*-direction as its surface normal. Because the peak splitting is independent of *q*_*xy*_, only *q*_*z*_ needs to be considered, which has the length of the difference between the *z*-components of the scattered (*k*_*z*_) and the incident wavevector (*k*_0*z*_). In order to further take into account the refraction by the organic adsorbate, its refractive index (*n*_org_) has to be considered (setting *n*_org_ = 1 would neglect refraction):
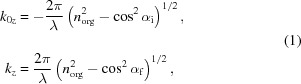
where λ is the primary-beam wavelength. As discussed above, diffraction by the organic layer arises either directly from the incident primary X-ray beam (peak A) or from the primary beam after its reflection at the substrate (peak A′). Therefore, it follows for the scattering vector in these two cases,
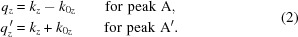
Fig. 5(*b*) ▸ compares the corrected values of *q*_*z*_ for the two peaks with the experimental values in reciprocal space. For peak A, the uncorrected values of *q*_*z*_ lie between 0.07 Å^−1^ and 0.065 Å^−1^, while considering refraction yields essentially identical values for all angles α_i_ with an average of *q*_*z*_ = 0.0594 Å^−1^. Following the Laue condition for diffraction, the *z*-component of the reciprocal lattice vector equals *q*_*z*_ and is independent of α_i_. On the basis of the single-crystal structure, the *z*-component of the 110 reciprocal lattice vector (*G*_110,*z*_) equals 0.0593 Å^−1^ for NNN, which is, therefore, in excellent agreement with the refraction-corrected *q*_*z*_ value. Analogously correcting the data of peak A′ both for the second scattering path and for refraction, we obtain values between 0.0655 Å^−1^ and 0.0529 Å^−1^ (average: *q*_*z*_ = 0.0602 Å^−1^). Here, the agreement between *q*_*z*_ and *G*_110,*z*_ is still good, although not perfect. Deviations are observed for α_i_ angles between 0.11° and 0.14°, since the peak A′ cannot be separated clearly from the dominant main peak. The deviations at large angles (α_i_ = 0.21, 0.22°) are explained by uncertainties of peak positions due to vanishing peak intensities. The deviations are within the calculated errors.

On the basis of the above explanation for the observed peak splitting, the expected angle between the two split peaks can now be predicted. Clearly, the Laue condition for the *z*-direction is valid for both X-ray paths, hence, also the respective *q*_*z*_ values have to be equal (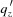 = *q*_*z*_),
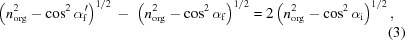
where 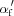 and α_f_ are the out-of-plane scattering angles of the two split peaks. If refraction is neglected, this equation would simplify (in good approximation for small angles) to: 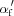 − α_f_ = 2α_i_. Refraction, however, becomes particularly important when the experiment is performed close to α_C_, notably, not only for α_i_ but also for α_f_ (and 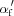) and, in principle, cannot be neglected in any GIXD experiment.

**Figure 6:**
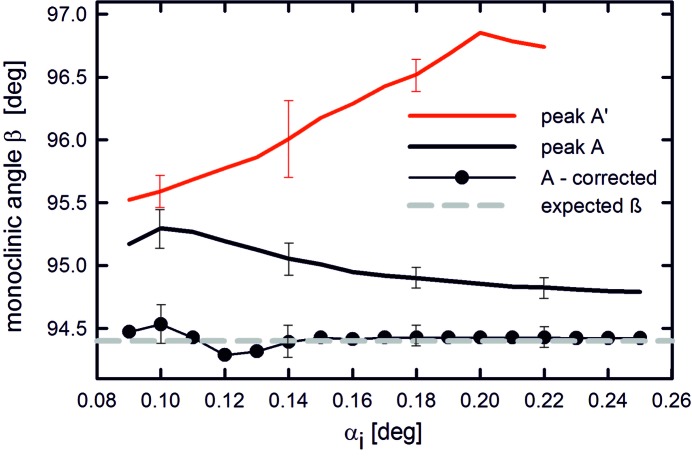
Monoclinic angle β of the NNN crystal structure as a function of the incident angle α_i_ as calculated from the peak positions of A′, A, and A after correction for refraction effects. Additionally, the monoclinic angle of the single-crystal structure solution is given; error bars consider only uncertainties in the peak positions of the out-of-plane scattering angle α_f_.

Frequently, reciprocal space maps of organic thin films are used as a basis for crystal-structure solution, where the initial, and maybe most important, step is the precise determination of the lattice constants as deduced from the positions of the individual Bragg peaks. The present experimental results on the NNN example show that a shift in the peak positions appears in the out-of-plane direction (*z* direction), while the peak positions in the in-plane directions (*x* and *y* directions) remain unaffected. The largest variations are observed when α_i_ and α_f_ are close to the critical angle of total reflection (α_C,substrate_) of the respective substrate material, as refraction effects and, therefore, the peak splitting resulting thereof are dominant under these conditions. In the present example of monoclinic NNN crystallites with their 001 planes parallel to the substrate surface, the lattice constants *a* and *b* determined by GIXD are *not* influenced by a shift of the Bragg peaks along *q*_*z*_. In contrast, the determination of both the lattice constant *c* and the monoclinic angle β are strongly affected. As an example, we present in Fig. 6 ▸ the derived monoclinic angle β compared with that expected from the single-crystal structure solution (Moret, 2015 ▸). To that end, we take into account the position of the 110 reflection which allows determination of β by using the lattice constants *a* and *b*, as taken from the known crystallographic unit cell. Fig. 6 ▸ shows the calculated values of β for the misleading cases, when peaks A′ and A were treated as being due to scattering of the primary beam at the 110 plane of NNN. For the case of peak A′, a large deviation from the expected value is obtained; for A, a smaller but still considerable deviation is found. If, however, the position of peak A is correctly treated for refraction effects (according to the formalism derived above), an excellent agreement with the expected value of β is found for all angles α_i_.

Broadened diffraction peaks can, however, cause considerable difficulties for the precise determination of lattice constants. In the *z*-direction, peak broadening can arise due to size and shape effects of the crystallites as well as due to peculiar primary/scattered beam-geometries. In the case of accordingly large peak widths, the split peaks can overlap to one single (artificial) peak where, therefore, the two scattering paths are no longer discriminable. Clearly, correcting the experimental peak positions for scattering path and refraction effects then becomes a major challenge. The impact of such unresolved effects on the derived lattice constants can be in the range of a few percent, which is large compared with lattice constants determined, for example, by single-crystal diffraction, where an accuracy of 10^−4^ is commonly achieved in standard investigations (Bennett, 2010 ▸).

## 4. Conclusions   

Grazing-incidence X-ray diffraction (GIXD) patterns of thin crystalline NNN films show all Bragg reflections doubled in the out-of-plane direction around the positions kinematically expected on the basis of the known crystal structure. The observed peak splitting is rationalized by two different scattering pathways of the incident X-rays, one of which leads to direct Bragg scattering at the organic crystallites, while the other is due to a two-step process described by an optically reflected primary beam at the substrate/organic interface and subsequent Bragg scattering at the organic layer. Because the peak splitting is observed only for α_i_ angles close to α_C_, optical refraction of X-rays within the organic material has to be taken into account to quantitatively correct for this effect. Simple mathematical expressions are provided for the correction of the experimental peak positions taking into account the very scattering paths as well as refraction effects. It is further demonstrated that the correction of the experimental peak positions allows the determination of the lattice constants of the crystalline thin-film structure with high accuracy. However, peak broadening effects can smear these double features rendering them unable to resolve. In such a case, choosing α_i_ values significantly beyond or below the angle of total external reflection of the substrate material α_C,substrate_ (*e.g.* 0.6α_C,substrate_ or 1.5α_C,substrate_) emerges as an experimental strategy for drastically reducing the effect, because the second scattering path then becomes strongly suppressed.
